# Supplementary material for: The causal relationship between the human gut microbiota and pyogenic arthritis: a Mendelian randomization study
Source: Front Cell Infect Microbiol. 2024 Nov 26;14:1452480. doi: 10.3389/fcimb.2024.1452480 (PMC11629706; doi:10.3389/fcimb.2024.1452480)
Supplement: Supplementary file 1 [file DataSheet1.zip › mendelian test/ebi-a-GCST90027751.csv_forest.pdf]

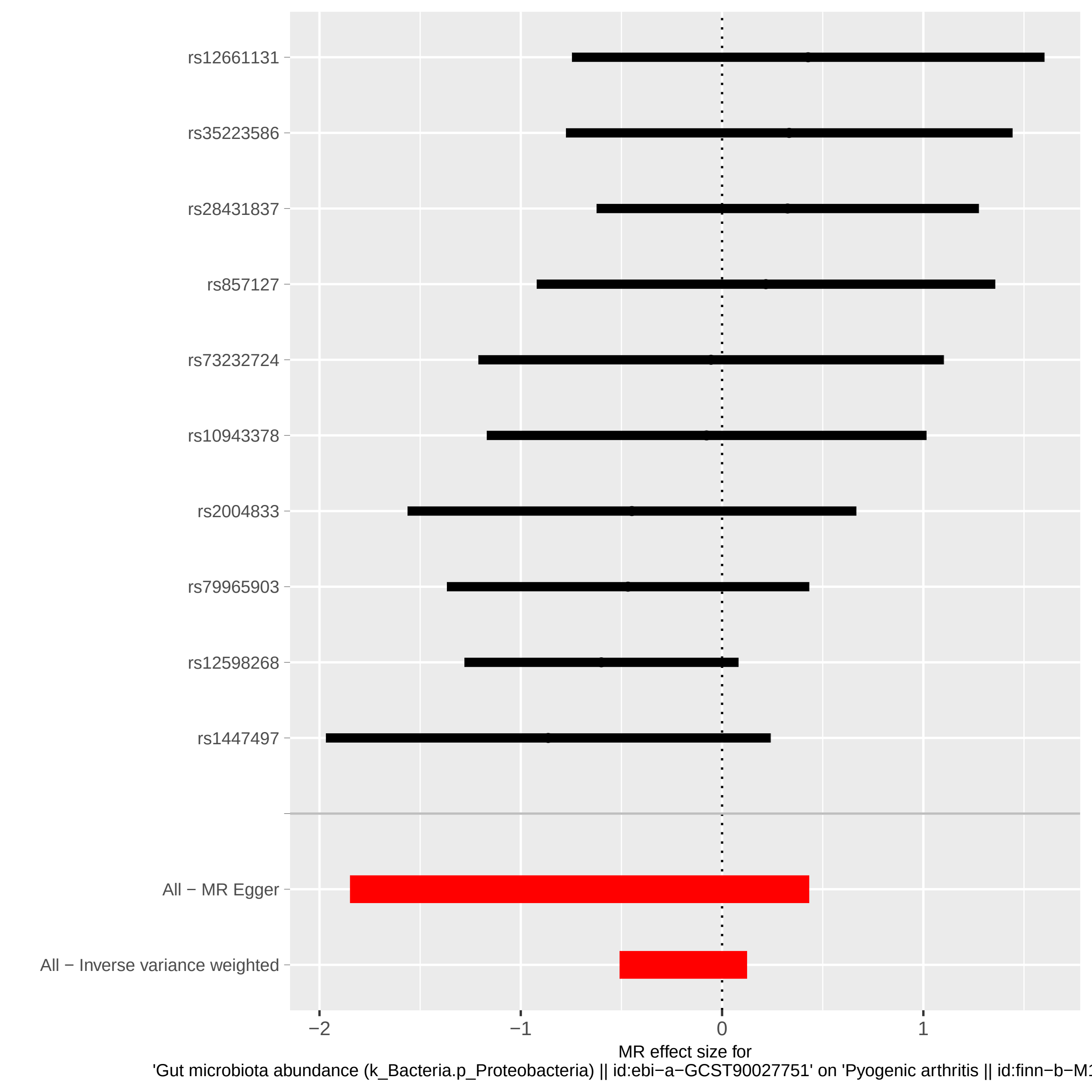

rs12661131
rs35223586
rs28431837
rs857127
rs73232724
rs10943378
rs2004833
rs79965903
rs12598268
rs1447497
All − MR Egger
All − Inverse variance weighted
−2
−1
0
1
MR effect size for
'Gut microbiota abundance (k_Bacteria.p_Proteobacteria) || id:ebi−a−GCST90027751' on 'Pyogenic arthritis || id:finn−b−M1
